# Supplementary material for: IGF1 and Insulin Receptor Single Nucleotide Variants Associated with Response in HER2-Negative Breast Cancer Patients Treated with Neoadjuvant Chemotherapy with or without a Fasting Mimicking Diet (BOOG 2013-04 DIRECT Trial)
Source: Cancers (Basel). 2023 Dec 17;15(24):5872. doi: 10.3390/cancers15245872 (PMC10742143; doi:10.3390/cancers15245872)
Supplement: Supplementary file 1 [file cancers-15-05872-s001.zip › DIRECT1-IGF1RINSR_manuscript_Tables-supplementary_final.pdf]

Supplementary Material

Table S1. Haploview

IGF1R SNV	Position	MAF	Alternative SNV	r^2	Genome position	MAF
rs66745311	99503083	0.24	rs1815009	0.92	99504671	0.24
rs3051367	99503521	0.43	rs2684788	0.94	99504437	0.42
rs9282714	99503690	0.20	rs2654980	1.00	99504843	0.20
**INSR SNV**						
rs2352955	7152404	0.22	rs2252673	1.00	7150418	0.22
rs34045095	7116136	0.24	None			
rs2352954	7152418	0.29	None			

Haploview selection tagger table of SNVs that are in equilibrium with the 6 IGF1R and INSR SNVs that were technically not suitable for assay, showing tagging results with SNVs r2 >0.8
SNV = single nucleotide variant, INSR = insulin receptor gene, IGF1R = insulin like growth factor 1 receptor, MAF = mean allele frequency

Table S2. SNV information

IGF1R 11 SNVs		MAF	Type of mutation	Ref and Alt allele		Clinical variation PubMed
1	rs3743258	0.303	Intron	G	A	
2	rs3743259	0.308	Intron	A	G	
3	rs2229765	0.419	Missense	G	A/T	Benign
4	rs45484096	0.268	Deletion, 3' prime UTR	CT	-	Benign
5	rs1815009	0.242	3' prime UTR	C	T	"
6	rs2684788	0.424	3' prime UTR	C	T	"
7	rs2654980	0.202	3' prime UTR	C	T	"
8	rs2016347	0.439	3' prime UTR	GAC	T	"
9	rs2684787	0.212	3' prime UTR	CA	T	"
10	rs2654981	0.419	3' prime UTR	CT	G	"
11	rs3743249	0.242	3' prime UTR	G	T	"
**INSR 6 SNVs**						
12	rs3745551	0.394	3' prime UTR	C	T	Benign
13	rs3833238	0.222	Indel, 3' prime UTR	T	-	Benign
14	rs1051651	0.207	3' prime UTR	G	C	Benign
15	rs1051690	0.242	3' prime UTR	C	T	Benign
16	rs1799817	0.268	Synonymous variant	A	G	Benign
17	rs2252673	0.222	Intron variant	C	G	Unknown

SNV = single nucleotide variant, INSR = insulin receptor gene, IGF1R = insulin like growth factor 1 receptor, MAF = mean allele frequency, Ref = reference allele, Alt = alternative SNV allele, UTR = untranslated region, Indel = in frame deletion

Table S3. Proportional odds assumption

A ***IGF1R* rs3743259 Odds Ratio**

	Miller & Payne score				
	**grade 1**	**grade 2**	**grade 3**	**grade 4**	**grade 5**
	no reduction	<30%	30-90%	>90%	no tumor
grade 1	Ref	0.424	0.429	0.145	0.267
grade 2	2.361	Ref	1.014	0.342	0.631
grade 3	2.329	0.986	Ref	0.337	0.622
grade 4	6.907	2.925	2.966	Ref	1.846
grade 5	3.742	1.585	1.607	0.542	Ref

B ***IGF1R* rs3743258 Odds Ratio**

	Miller & Payne score				
	**grade 1**	**grade 2**	**grade 3**	**grade 4**	**grade 5**
	no reduction	<30%	30-90%	>90%	no tumor
grade 1	Ref	0.386	0.373	0.125	0.265
grade 2	2.592	Ref	0.966	0.323	0.686
grade 3	2.684	1.035	Ref	0.335	0.71
grade 4	8.016	3.093	2.987	Ref	2.121
grade 5	3.78	1.458	1.408	0.472	Ref

Odds ratios for IGF1R rs3743259 (**A)** and IGF1R rs3743258 (**B**)

Table S4. SNV information

**SNV**	**No call**	**Call rate**	**MAF Reference Allele**	**MAF SNV Allele**	**1000Genomes MAF SNV Allele**	**PubMed**	**HWE**	**P-value***
IGF1R rs2016347	0	100.0%	53%	47%	0.57	0.49	0.003	0.957
IGF1R rs2229765	1	99.1%	58%	42%	0.42	0.45	1.116	0.291
IGF1R rs1815009	0	100.0%	26%	74%	0.75	0.75	1.735	0.188
INSR rs1051651	1	99.1%	82%	18%	0.21	0.18	2.447	0.118
INSR rs3745551	1	99.1%	33%	67%	0.60	0.64	0.009	0.924
IGF1R rs3743259	2	98.2%	71%	29%	0.31	0.29	1.297	0.255
IGF1R rs2684787	1	99.1%	72%	28%	0.21	0.22	0.449	0.503
IGF1R rs2654981	0	100.0%	46%	54%	0.42	0.39	0.053	0.818
IGF1R rs2654980	0	100.0%	73%	27%	0.20	0.23	0.499	0.480
IGF1R rs2684788	0	100.0%	48%	52%	0.42	0.46	0.072	0.788
IGF1R rs3743249	0	100.0%	73%	27%	0.43	0.25	0.346	0.556
IGF1R rs45484096	0	100.0%	65%	35%	0.27	0.31	1.118	0.290
INSR rs3833238	0	100.0%	81%	19%	0.22	0.19	1.700	0.192
INSR rs1051690	0	100.0%	15%	85%	0.76	0.84	0.095	0.757
INSR rs1799817	0	100.0%	86%	14%	0.27	0.18	1.802	0.179
INSR rs2252673	5	95.6%	13%	87%	0.78	0.81	0.760	0.383
IGF1R rs3743258	9	92.0%	72%	28%	0.30	0.29	3.072	0.080

*(if < 0.05 - not consistent with HWE); Call-rate minimum is set at 85%.
SNV single nucleotide variant, INSR insulin receptor, IGF1R insulin like growth factor 1 receptor, MAF mean allele frequency, HWE Hardy-Weinberg equation.

Table S5. Univariate model for covariates

			Miller & Payne (n=114)				Radiological response (n=96)			
**A**	**Variable**		**OR**	**95% CI lower-upper**		**p-value**	**OR**	**95% CI lower-upper**		**p-value**
	Age		1.00	0.96	1.04	0.87	1.04	0.98	1.09	0.20
	BMI		0.98	0.91	1.05	0.54	1.05	0.96	1.15	0.31
	Tumor status		2.22	1.21	4.07	**0.01**	0.58	0.27	1.24	0.16
	Lymph node status		0.44	0.26	0.75	**0.003**	1.06	0.57	1.98	0.86
	Tumor type	Other	0.06	0.00	0.75	**0.03**	1.63	0.13	19.77	0.70
		Lobular	0.46	0.18	1.16	0.10	1.22	0.37	4.06	0.75
		Ductal/Carcinoma	2.72	Ref			2.72	Ref		
	HR-status	ER-/PR-	0.52	0.18	1.49	0.22	0.93	0.25	3.40	0.91
		ER+/PR-	0.77	0.24	2.45	0.65	1.75	0.45	6.77	0.42
		ER+/PR+	2.72	Ref			2.72	Ref		
	Treatment	FMD	0.96	0.48	1.92	0.92	0.71	0.29	1.73	0.46
		Regular diet	2.72	Ref			2.72	Ref		

			Miller & Payne (n=74)				Radiological response (n=65)			
**B**	**Variable**		**OR**	**95% CI lower-upper**		**p-value**	**OR**	**95% CI lower-upper**		**p-value**
	Age		1.02	0.96	1.08	0.56	1.02	0.96	1.09	0.53
	BMI		1.00	0.91	1.11	0.95	1.05	0.93	1.18	0.45
	Tumor status		0.48	0.23	0.99	**0.05**	0.59	0.25	1.39	0.23
	Lymph node status		1.87	0.98	3.56	0.06	1.34	0.66	2.71	0.41
	Tumor type	Other	$1.74*10^{9}$	$1.74*10^{9}$	$1.74*10^{9}$					
		Lobular	2.23	0.76	6.57	0.14	1.30	0.37	4.57	0.68
		Ductal/Carcinoma	2.72	Ref	Ref		2.72	Ref	Ref	
	HR-status	ER-/PR-	2.61	0.46	14.92	0.28	0.38	0.04	3.16	0.37
		ER+/PR-	0.88	0.22	3.60	0.86	1.12	0.22	5.70	0.89
		ER+/PR+	2.72	Ref	Ref		2.72	Ref	Ref	
	Treatment	FMD compliant	0.51	0.19	1.39	0.19	0.47	0.15	1.49	0.20
		Regular diet	2.72	Ref	Ref		2.72	Ref	Ref	

A ITT univariate model for covariates. **B** PP univariate model for covariates

ITT intention-to-treat, PP per protocol, FMD fasting mimicking diet, OR odds ratio, CI confidence interval, BMI body mass index (kg/m2), HR-status hormone receptor status, ER estrogen receptor, PR progesterone receptor

Table S6. Logistic regression models responders versus non-responders

A	ITT	Miller & Payne 1-3 vs 4-5				Radiological response CR/PR vs SD/PD			
	**SNVs**	**OR**	**95% CI lower-upper**		**p-value**	**OR**	**95% CI lower-upper**		**p-value**
	IGF1R rs2016347	1.37	2.03 -	13.91	0.35	0.63	1.31 -	4.29	0.28
	IGF1R rs2229765	1.14	1.75 -	10.06	0.72	0.98	1.52 -	9.69	0.96
	IGF1R rs1815009	1.39	1.80 -	27.05	0.45	1.11	1.54 -	16.74	0.83
	INSR rs1051651	1.31	1.83 -	17.22	0.49	1.87	2.13 -	99.88	0.17
	INSR rs3745551	1.06	1.70 -	8.31	0.87	2.24	2.32 -	381.84	0.11
	IGF1R rs3743259	0.35	1.16 -	2.33	0.02	0.79	1.40 -	6.40	0.59
	IGF1R rs2684787	1.48	2.10 -	19.63	0.27	0.76	1.37 -	6.22	0.54
	IGF1R rs2654981	1.13	1.81 -	8.55	0.71	0.73	1.39 -	5.07	0.44
	IGF1R rs2654980	1.49	2.10 -	19.78	0.26	0.72	1.35 -	5.55	0.45
	IGF1R rs2684788	1.29	1.96 -	11.95	0.44	0.79	1.43 -	5.70	0.55
	IGF1R rs3743249	0.94	1.53 -	7.78	0.87	0.79	1.38 -	7.12	0.62
	IGF1R rs45484096	1.31	1.99 -	12.20	0.41	0.80	1.43 -	5.99	0.58
	INSR rs3833238	1.32	1.86 -	16.64	0.47	2.10	2.33 -	179.83	0.11
	INSR rs1051690	0.38	1.16 -	2.59	0.039	1.43	1.59 -	84.44	0.534
	INSR rs1799817	0.83	1.39 -	8.28	0.70	2.63	2.23 -	5585.90	0.11
	INSR rs2252673	0.82	1.36 -	8.92	0.69	2.21	1.72 -	7871.47	0.27
	IGF1R rs3743258	0.36	1.15 -	2.45	0.03	0.81	1.41 -	6.65	0.63
**B**	**PP**	**Miller & Payne 1-3 vs 4-5**				**Radiological response CR/PR vs SD/PD**			
	**SNVs**	**OR**	**95% CI lower-upper**		**p-value**	**OR**	**95% CI lower-upper**		**p-value**
	IGF1R rs2016347	1.18	1.65 -	16.30	0.70	0.55	1.19 -	5.40	0.29
	IGF1R rs2229765	1.09	1.57 -	13.83	0.85	0.76	1.27 -	11.20	0.65
	IGF1R rs1815009	2.04	2.03 -	355.06	0.19	0.54	1.18 -	5.62	0.30
	INSR rs1051651	0.86	1.41 -	8.94	0.76	1.77	1.70 -	362.78	0.35
	INSR rs3745551	2.11	2.31 -	199.45	0.11	2.31	2.01 -	2135.46	0.17
	IGF1R rs3743259	0.59	1.26 -	4.50	0.27	1.13	1.48 -	25.74	0.83
	IGF1R rs2684787	1.74	2.00 -	77.48	0.24	0.36	1.11 -	3.68	0.12
	IGF1R rs2654981	0.92	1.46 -	9.23	0.85	0.68	1.25 -	7.83	0.49
	IGF1R rs2654980	1.75	2.01 -	79.56	0.23	0.33	1.10 -	3.29	0.09
	IGF1R rs2684788	1.05	1.57 -	11.61	0.90	0.64	1.24 -	6.66	0.42
	IGF1R rs3743249	0.61	1.25 -	5.23	0.33	1.45	1.62 -	81.21	0.51
	IGF1R rs45484096	1.50	1.93 -	30.95	0.33	0.50	1.18 -	4.71	0.23
	INSR rs3833238	0.87	1.41 -	9.07	0.78	1.85	1.69 -	669.22	0.34
	INSR rs1051690	0.25	1.07 -	2.44	0.033	1.51	1.40 -	825.77	0.588
	INSR rs1799817	0.33	1.07 -	4.60	0.16	2.10	1.47 -	$9.46*10^4$	0.39
	INSR rs2252673	1.33	1.50 -	76.88	0.64	2.17	1.45 -	$3,59*10^5$	0.39
	IGF1R rs3743258	0.64	1.28 -	5.17	0.35	1.17	1.50 -	29.53	0.77

A Intention-to-treat logistic regression analysis of IGF1R and INSR SNVs with clinical responders and non-responders. **B** Per-protocol logistic regression analysis of IGF1R and INSR SNVs with clinical responders and non-responders.
Cut off value Bonferroni correction for multiple testing is 0.05/17 =0.0029
OR odds ratio, CI confidence interval, SNV single nucleotide variant, INSR insulin receptor, IGF1R insulin like growth factor 1 receptor. CR complete response. PR partial response. SD stable disease. PD progression of disease.

Table S7. Generalized linear model SNVs and treatment interaction

A **ITT Interaction FMD and SNV**

	Miller & Payne					Radiological response				
**SNVs**	**OR**	**95% CI lower-upper**			**p-value**	**OR**	**95% CI lower-upper**			**p-value**
IGF1R rs2016347	1.31	0.47	-	3.68	0.61	1.67	0.45	-	6.10	0.44
IGF1R rs2229765	1.03	0.34	-	3.13	0.95	1.43	0.36	-	5.63	0.61
IGF1R rs1815009	0.98	0.29	-	3.23	0.97	3.72	0.78	-	17.70	0.10
INSR rs1051651	1.01	0.31	-	3.25	0.99	1.22	0.26	-	5.83	0.80
INSR rs3745551	0.68	0.25	-	1.87	0.45	0.38	0.10	-	1.50	0.17
IGF1R rs3743259	0.48	0.17	-	1.40	0.18	1.15	0.31	-	4.31	0.83
IGF1R rs2684787	2.14	0.65	-	7.05	0.21	5.13	1.12	-	23.63	0.036
IGF1R rs2654981	2.03	0.74	-	5.55	0.17	1.28	0.35	-	4.63	0.71
IGF1R rs2654980	2.02	0.62	-	6.61	0.25	5.71	1.26	-	25.85	0.024
IGF1R rs2684788	1.91	0.69	-	5.30	0.21	1.48	0.42	-	5.27	0.54
IGF1R rs3743249	0.95	0.30	-	2.97	0.93	0.49	0.12	-	2.03	0.32
IGF1R rs45484096	1.58	0.55	-	4.50	0.39	2.35	0.59	-	9.31	0.22
INSR rs3833238	0.98	0.31	-	3.09	0.97	1.76	0.36	-	8.67	0.49
INSR rs1051690	0.89	0.22	-	3.59	0.872	0.27	0.04	-	1.76	0.172
INSR rs1799817	1.33	0.34	-	5.27	0.68	0.75	0.09	-	6.20	0.79
INSR rs2252673	0.26	0.05	-	1.30	0.10	3.13	0.40	-	24.70	0.28
IGF1R rs3743258	0.44	0.15	-	1.34	0.15	1.22	0.33	-	4.56	0.77

B **PP Interaction FMD and SNV**

	Miller & Payne					Radiological response				
**SNVs**	**OR**	**95% CI lower-upper**			**p-value**	**OR**	**95% CI lower-upper**			**p-value**
IGF1R rs2016347	1.50	0.40	-	5.64	0.55	2.34	0.44	-	12.42	0.32
IGF1R rs2229765	2.21	0.58	-	8.52	0.25	1.54	0.29	-	7.99	0.61
IGF1R rs1815009	2.13	0.44	-	10.37	0.35	1.24	0.19	-	8.00	0.82
INSR rs1051651	0.45	0.11	-	1.78	0.25	1.34	0.24	-	7.33	0.74
INSR rs3745551	1.72	0.49	-	6.03	0.40	0.21	0.04	-	1.06	0.06
IGF1R rs3743259	0.42	0.09	-	2.02	0.28	1.72	0.34	-	8.65	0.51
IGF1R rs2684787	4.21	0.98	-	18.02	0.05	2.86	0.45	-	18.32	0.27
IGF1R rs2654981	2.22	0.61	-	8.11	0.23	1.71	0.34	-	8.54	0.51
IGF1R rs2654980	3.95	0.93	-	16.88	0.06	2.98	0.47	-	19.02	0.25
IGF1R rs2684788	2.20	0.61	-	7.98	0.23	1.82	0.38	-	8.70	0.45
IGF1R rs3743249	0.38	0.08	-	1.83	0.23	1.17	0.19	-	7.23	0.86
IGF1R rs45484096	2.81	0.75	-	10.54	0.12	1.72	0.33	-	8.91	0.52
INSR rs3833238	0.53	0.13	-	2.13	0.37	1.48	0.26	-	8.45	0.66
INSR rs1051690	0.34	0.04	-	2.66	0.302	0.27	0.02	-	3.31	0.309
INSR rs1799817	0.55	0.09	-	3.41	0.52	0.64	0.06	-	7.44	0.72
INSR rs2252673	0.41	0.06	-	2.90	0.37	4.47	0.45	-	44.37	0.20
IGF1R rs3743258	0.30	0.05	-	1.74	0.18	1.52	0.29	-	7.94	0.62

A Intention-to-treat GLM model **B** Per-protocol GLM model

Cut off value Bonferroni correction for multiple testing is 0.05/17 =0.0029

GLM generalized linear model, ITT intention-to-treat, PP per protocol, OR odds ratio, CI confidence interval, SNV single nucleotide variant, INSR insulin receptor gene, IGF1R insulin like growth factor 1 receptor. CR complete response. PR partial response. SD stable disease. PD progression of disease.

Table S8. Generalized linear model SNVs and treatment interaction responders versus non-responders

A ITT Interaction FMD and SNV

SNVs	OR	Miller & Payne 1-3 vs 4-5<br>95% CI lower-upper			p-value	OR	Radiological response CR/PR vs SD/PD<br>95% CI lower-upper			p-value
IGF1R rs2016347	1.24	0.31	-	4.97	0.76	0.85	0.16	-	4.59	0.85
IGF1R rs2229765	1.39	0.31	-	6.21	0.66	1.16	0.21	-	6.35	0.87
IGF1R rs1815009	1.12	0.21	-	6.09	0.89	10.28	1.08	-	98.03	0.043
INSR rs1051651	0.43	0.08	-	2.24	0.32	1.22	0.19	-	7.69	0.83
INSR rs3745551	0.99	0.24	-	4.19	0.99	1.33	0.19	-	9.08	0.77
IGF1R rs3743259	0.26	0.04	-	1.50	0.13	0.54	0.09	-	3.07	0.48
IGF1R rs2684787	1.94	0.42	-	8.94	0.39	3.22	0.51	-	20.15	0.21
IGF1R rs2654981	1.68	0.40	-	7.02	0.48	0.66	0.13	-	3.29	0.61
IGF1R rs2654980	1.93	0.42	-	8.88	0.40	3.62	0.58	-	22.63	0.17
IGF1R rs2684788	1.97	0.49	-	8.01	0.34	0.96	0.19	-	4.69	0.96
IGF1R rs3743249	0.92	0.20	-	4.31	0.92	0.16	0.02	-	1.41	0.10
IGF1R rs45484096	1.28	0.32	-	5.04	0.73	2.00	0.36	-	11.15	0.43
INSR rs3833238	0.38	0.08	-	1.85	0.23	1.94	0.27	-	13.93	0.51
INSR rs1051690	1.52	0.23	-	10.09	0.665	0.40	0.05	-	3.52	0.409
INSR rs1799817	2.78	0.23	-	33.69	0.42	1.37	0.11	-	16.66	0.80
INSR rs2252673	0.17	0.02	-	1.63	0.12	1.36	0.07	-	27.61	0.84
IGF1R rs3743258	0.21	0.03	-	1.30	0.09	0.63	0.11	-	3.51	0.60

B PP Interaction FMD and SNV

SNVs	OR	Miller & Payne<br>95% CI lower-upper			p-value	OR	Radiological response<br>95% CI lower-upper			p-value
IGF1R rs2016347	0.94	0.17	-	5.28	0.94	0.69	0.05	-	9.52	0.78
IGF1R rs2229765	1.92	0.32	-	11.63	0.48	1.20	0.09	-	16.14	0.89
IGF1R rs1815009	1.59	0.44	-	53.58	0.79	4.36	0.25	-	75.78	0.31
INSR rs1051651	0.07	0.01	-	0.70	0.024	0.88	0.08	-	9.99	0.92
INSR rs3745551	5.24	0.73	-	37.80	0.10	0.99	0.07	-	14.06	0.99
IGF1R rs3743259	0.77	0.10	-	5.80	0.80	1.50	0.13	-	16.89	0.74
IGF1R rs2684787	4.51	0.52	-	39.39	0.17	1.49	0.08	-	27.82	0.79
IGF1R rs2654981	1.44	0.24	-	8.58	0.69	0.66	0.06	-	7.82	0.74
IGF1R rs2654980	4.50	0.51	-	39.38	0.17	1.58	0.08	-	30.05	0.76
IGF1R rs2684788	1.55	0.29	-	8.29	0.61	0.67	0.06	-	8.01	0.75
IGF1R rs3743249	0.16	0.01	-	1.76	0.13	0.31	0.02	-	5.45	0.43
IGF1R rs45484096	1.92	0.33	-	11.01	0.47	0.93	0.05	-	15.69	0.96
INSR rs3833238	0.07	0.01	-	0.69	0.023	0.81	0.06	-	10.06	0.87
INSR rs1051690	0.48	0.03	-	9.16	0.629	0.08	0.00	-	3.44	0.185
INSR rs1799817	0.50	0.02	-	11.46	0.66	0.50	0.02	-	14.74	0.69
INSR rs2252673	0.40	0.03	-	4.89	0.47	$1.35*10^9$	0	-	infinity	1.00
IGF1R rs3743258	0.51	0.05	-	4.67	0.55	1.44	0.13	-	16.25	0.77

A Intention-to-treat GLM model **B** Per-protocol GLM model

Cut off value Bonferroni correction for multiple testing is 0.05/17 =0.0029

GLM generalized linear model, ITT intention-to-treat, PP per protocol, OR odds ratio, CI confidence interval, SNV single nucleotide variant, INSR insulin receptor, IGF1R insulin like growth factor 1 receptor. CR complete response. PR partial response. SD stable disease. PD progression of disease.
